# Psychotherapy of women victims of domestic violence: Ukrainian practice

**DOI:** 10.1192/j.eurpsy.2025.818

**Published:** 2025-08-26

**Authors:** M. Markova, I. Romanova, A. Avramenko, S. Martynenko, V. Pliekhov, T. Aliieva

**Affiliations:** 1 Sexology, Psychotherapy and Medical Psychology, Kharkiv National Medical University, Kharkiv, Ukraine

## Abstract

**Introduction:**

According to the press service of the National Police of Ukraine, the number of domestic violence complaints in 2023 amounted to 291,000 statements from victims, which is almost 2 times more than in 2022. This trend determines the relevance of improving the model of comprehensive assistance to victims of domestic violence in Ukraine.

**Objectives:**

To develop and study the effectiveness of a comprehensive system of psychotherapy for women victims of domestic violence.

**Methods:**

We have been examined 85 women victims of domestic violence during 2022-2023; 59 % of them were suffered from physical violence, 100 % – psychological violence, 6 % - economic violence. The following methods were used: Spielberger-Y.L.Khanin scale of reactive and personal anxiety (STAI), Eysenck Personality Questionnaire (EPQ) to determine the level of neuroticism, The Thomas–Kilmann Conflict Mode Instrument (TKI), The Hamilton Depression Rating Scale (HDRS).

**Results:**

The most common form of psychological violence against women was manipulation of the child’s interests during divorce proceedings, when men tried to insult and humiliate the authority of their wives, displaying aggressive forms of behavior. Most aggressors are characterized by emotional instability, irritability and cruelty. An aggravating psychological factor in conflicts in such families was the abuse of alcohol by men.

Women who suffered from domestic violence showed psychopathological personality changes not only in the form of victim character traits, but also in the form of aggression. Other women, on the contrary, were passive, conformist, and could not protect themselves. We used the following methods of psychotherapy: telephone and online counseling, psychological counseling, behavioral psychotherapy, systemic family psychotherapy, and rational psychotherapy. Psychological counseling solved the problems of resolving the difficulties of women victims by creating conditions for expressing strong emotions and helping them gain a sense of control over themselves. The comprehensive system of psychotherapy was aimed at assessing the psychotraumatic situation of a case of violence and forming new ideas about family life and developing new reactions and forms of behavior, forming victim personality traits in women.

**Conclusions:**

The comprehensive system of psychotherapy for women victims of domestic violence was developed. As a result, 82% of women who received the indicated therapy experienced a decrease in manifestations of neurotic and somatoform syndromes, an increase in self-esteem, self-confidence, an improvement in the well-being of women, and an improvement in the psychological climate in these families.

**Disclosure of Interest:**

None Declared

